# Botulinum Toxin Type A—A Modulator of Spinal Neuron–Glia Interactions under Neuropathic Pain Conditions

**DOI:** 10.3390/toxins10040145

**Published:** 2018-04-02

**Authors:** Ewelina Rojewska, Anna Piotrowska, Katarzyna Popiolek-Barczyk, Joanna Mika

**Affiliations:** Department of Pain Pharmacology Institute of Pharmacology, Polish Academy of Sciences, 31-343 Krakow, Poland; rojewska@if-pan.krakow.pl (E.R.); anna.piotrowskamurzyn@gmail.com (A.P.); popiolek@if-pan.krakow.pl (K.P.-B.)

**Keywords:** BoNT/A, astroglia, interleukins, microglia, TLR2, TLR4, Snap-23

## Abstract

Neuropathic pain represents a significant clinical problem because it is a chronic condition often refractory to available therapy. Therefore, there is still a strong need for new analgesics. Botulinum neurotoxin A (BoNT/A) is used to treat a variety of clinical diseases associated with pain. Glia are in continuous bi-directional communication with neurons to direct the formation and refinement of synaptic connectivity. This review addresses the effects of BoNT/A on the relationship between glia and neurons under neuropathic pain. The inhibitory action of BoNT/A on synaptic vesicle fusion that blocks the release of miscellaneous pain-related neurotransmitters is known. However, increasing evidence suggests that the analgesic effect of BoNT/A is mediated through neurons and glial cells, especially microglia. In vitro studies provide evidence that BoNT/A exerts its anti-inflammatory effect by diminishing NF-κB, p38 and ERK1/2 phosphorylation in microglia and directly interacts with Toll-like receptor 2 (TLR2). Furthermore, BoNT/A appears to have no more than a slight effect on astroglia. The full activation of TLR2 in astroglia appears to require the presence of functional TLR4 in microglia, emphasizing the significant interaction between those cell types. In this review, we discuss whether and how BoNT/A affects the spinal neuron–glia interaction and reduces the development of neuropathy.

## 1. The Therapeutic Effect of Bont/A—Powerful Analgesic Agent against Neuropathic Pain

Neuropathic pain is caused by damage or injury to the nerves, and it is commonly experienced in the human and animal populations, involving a huge number of pathological conditions. Recent clinical and experimental research has shown that neuroimmune factors significantly influence the modulation of the pain development process [1,2,3,4]. The mechanism of the development and persistence of chronic pain remains an important clinical problem. Current knowledge does not allow us to fully assess the process and identify its most crucial elements. As suggested by data available from the International Association for the Study of Pain, one out of every five Europeans suffers from chronic pain of various origins and must make lifestyle changes for that reason. An additional problem is posed by the lack of appropriate therapy for these disorders. The treatment of neuropathic pain is a therapeutic challenge, and many pharmacological and non-pharmacological interventions have been suggested, with unsatisfying results [5,6,7,8,9]. Pharmacological medicaments include non-steroidal anti-inflammatory drugs, antidepressants, anticonvulsants, and opioids [6]. However, their use increases the risk of adverse events, as well as reductions in analgesic efficacy. The mechanism of this loss in efficacy is also not sufficiently understood to fully counteract this phenomenon and effectively treat pain. More effective treatments based on new targets and mechanisms of action are still being sought. This is the case for botulinum toxin A (BoNT/A), the use and application of which is increasing and becoming more widespread with time. BoNT/A is one of seven different BoNT types originating from *Clostridium botulinum* and is the most poisonous substance known to man [10], but, paradoxically, it has been widely used in the clinic, and its importance as a therapeutic agent is rising [11,12,13,14,15,16,17,18]. The well-defined mechanism of BoNT/A action is based on preventing release of the neurotransmitter acetylcholine from presynaptic nerve terminals through fragmentation of the protein SNAP-25 [19]. BoNT/A inhibits the release not only of acetylcholine but also of other neurotransmitters and neuropeptides, such as substance P and the calcitonin gene-related peptide (CGRP) [20,21,22]. The toxin also blocks conductivity in the autonomic system through sensory fibers and reduces the majority of substances acting on nociceptors [23]. Therefore, scientists are using it for clinical applications, including therapy for neuropathic pain. 

BoNT/A has been used in medical practice since 1989, when it was first included in the medication list by The Food and Drug Administration in the United States. A year later, the report of the American Academy of Neurology stated that BoNT/A injections are efficient in treating blepharospasms, facial spasticity, tremor, hyperhidrosis, hypersalivation, and wrinkle correction [24]. The evidence for the efficacy of BoNT/A in neuropathic pain relief in humans was first presented by Klein in 2004 [25] in relation to neuropathic pain linked to multiple sclerosis, neuralgia and peripheral neuropathy. BoNT/A has been successfully used in clinical practice for the treatment of many types of headaches [16,26], migraine [14,27,28], arthritic pain [29], cerebral palsy with serious acute sialadenitis [29], and recently also in small-fiber neuropathy [30], trigeminal neuralgia [11,17,31] and refractory joint pain [18]. Presently, BoNT/A usage has been extended to many medical conditions, including in urological, gastroenterological, and surgical contexts [32]. BoNT/A is often used to reduce spasticity in a neurorehabilitation setting and to treat other disorders, including complex regional pain syndrome with focal dystonia and phantom limb [12,13]. Many papers suggest that the use of BoNT/A represents a novel therapeutic strategy, especially for neuropathic pain, whenever widely use pharmacological agents have been ineffective [29,30,33]. Animal studies, including ours, support the clinical observations [31,34,35,36,37,38,39]. Interestingly, BoNT/A also increases morphine-induced analgesia and prevents the development of morphine tolerance upon long-term treatment [40,41]. Despite its wide application in medicine, its mechanisms of action are still not fully understood. Several lines of evidence indicate the crucial role of neuron–glia interactions in the development and progression of neuropathic pain. Since an analgesic action of BoNT/A has been demonstrated, the question concerning the role of this toxin in the modulation of neuron–glia interactions arises. This review addresses the effects of BoNT/A on the relationship between glial and neuronal cells in treating neuropathic pain.

## 2. Mechanism-Based Evidence for the Analgesic Actions of Bont/A

The peripheral sensory neurons conduct nociceptive information, which enters the spinal cord dorsal horn and then from the spinal projection is conveyed to supraspinal structures (such as the brainstem, thalamus, somatosensory cortex, insular cortex and anterior cingulate cortex) via ascending pathways. Nerve injury form pre- and postsynaptic long-term plasticity within the brain structures, which contributes to emotional and motivational aspects of neuropathic pain condition. Studies using human brain imaging and genetically modified mice have shown that neuropathy is largely due to long-term plastic changes within the sensory pathways [42,43]. In the nervous system, neuronal activity is mediated by synaptic release of neurotransmitters. Under resting conditions, synaptic vesicles are delivered to plasma membrane and may undergo constitutive exocytosis after fusion with newly synthesized proteins in the membrane without uniquely identifiable regulators [44,45,46]. In contrast to constitutive the regulated exocytosis is under the strict control of calcium signals present only in activated neurons experiencing a rise in calcium concentration. During this process, the probability of vesicle fusion rises dramatically by increasement of cytosolic calcium level, which is caused by the opening of voltage-gated calcium channels in response to the presence of an action potential at a nerve terminal [19,47,48]. In vivo studies showed that fast-regulated exocytosis requires the interaction of a members of protein superfamily, called SNAREs (soluble *N*-ethylmaleimide-sensitive factor attachment protein receptors), which are small cytoplasmically exposed membrane proteins. In regulated exocytosis, relevant SNAREs include synaptobrevin/VAMP (located on the membrane of the vesicle) and syntaxin-1 and SNAP-25 and/or its analogue SNAP-23 (localized on the plasma membrane), which create a complex representing the minimal mechanism required for fusion [49]. Increased calcium concentration (caused by calcium influx through voltage-gated calcium channels), is detected by synaptotagmin, and triggers fusion of docked synaptic vesicles, resulting in neurotransmitter release. Fusion is driven by a progressive zippering of vesicle and SNAREs proteins to form a strong four-helix bundle. Thus, the assembly of a SNARE complex represents crucial steps for nociceptive transmission between neuronal cells in the presence of neuropathic pain. Blockage of synaptic neurotransmission is strictly due to an inhibition of neurotransmitter release via vesicle-regulated exocytosis, and BoNT/A represents a powerful tool for the investigation of the involvement of SNAREs in exocytosis [35,50].

The involvement of BoNT/A in pain modulation mechanisms was first described by Cui et al., 2004 [51], who studied the effects of this toxin in inflammatory pain caused by formalin administration. Acting via SNAP-25, BoNT/A strongly inhibits the neuronal release of neurotransmitters and neuropeptides involved in nociceptive transmission, such as glutamate [51], substance P [20] and CGRP [21,52]. The authors demonstrated that single subcutaneous administration of BoNT/A into the paw after formalin injection reduced paw swelling and hypersensitivity to pain, and this effect was associated with inhibition of pronociceptive factors. In 2006, Luvisetto et al. [53], using a murine model of inflammatory pain, demonstrated that BoNT/A may act not only on the peripheral but also on the central nervous system (CNS). The analgesic effect of BoNT/A was also demonstrated in other inflammatory pain models, such as administration of carrageenan and capsaicin into the paw [34]. The authors suggested that intraarticular administration of BoNT/A is a promising method for the treatment of arthritis. The effects of BoNT/A were also studied in visceral pain models; it was also shown that intravenous administration of BoNT/A caused analgesia in an acetic acid-induced bladder pain model in rats [54]. In 2008, Antonucci et al. [55] suggested that the effects of BoNT/A might be due to retrograde transport or to the effect of transcytosis. Beneficial effects of BoNT/A were also observed in numerous clinical studies under neuropathic pain [11,12,13,14,15,16,17,18,30,31,38,39,56,57]. Several studies, including those conducted by our group, have shown that intraplantar injections of BoNT/A are also effective for treating neuropathic pain [31,35,36,38,40,58]. The results showed that BoNT/A diminished neuropathic pain by suppressing the secretion of neurotransmitters from neurons. Interesting results were obtained by the group of Vacca [40,41], who showed that administration of BoNT/A increased morphine antinociceptive action and countered morphine-induced tolerance after chronic treatment. These data suggest that BoNT/A is not only a potent analgesic but might also be useful as a component of multimodal pain therapy. 

Attention has been paid to older works showing that radiolabeled BoNT/A injected into the cat gastrocnemius muscle is detected in the spine 24–38 h after administration [59,60]. Antonucci et al. [55] suggested retrograde transport and transcytosis as a mechanism by which BoNT/A acts not only at the administration site but also at the distant areas that project to the infusion region. In 2012, Marinelli et al. [61] used a murine model of neuropathy to demonstrate that BoNT/A injected intraplantarly may migrate from the site of administration into the sciatic nerve, DRG and spinal cord. Evidenced provided by Cui et al. [51] showed that analgesic BoNT/A action is correlated with modulation of central sensitization process, while BoNT/A has no impact on acute pain. Proposed mechanism of BoNT/A action is that toxin inhibits the release of neurotransmitters from peripheral nerve endings and reduces peripheral sensitization. Following this process, the afferent input to the spinal cord is damped and central sensitization is reduced, which suggest indirectly role of BoNT/A in this process. However, direct mechanism is also possible, as it was already shown that BoNT/A might be transported via retrograde transport along the nociceptive neurons [55]. Based on those hypotheses, peripherally applied BoNT/A gains access to CNS and directly inhibits neurotransmitters release onto dorsal horn neurons.

**Figure 1:**
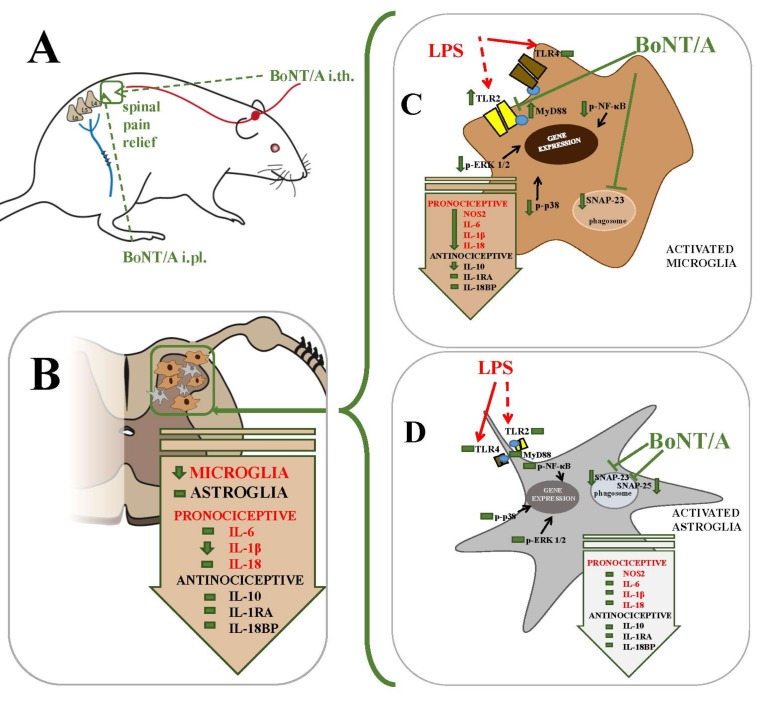
Suggested mechanism of botulinum toxin type A action under neuropathic pain conditions. (**A**) Intrathecal (*i.th.*) as well as intraplantar (*i.pl.*) injections of BoNT/A relief pain in animal models of neuropathy. Recent evidence suggests the possible retrograde transport and transcytosis of BoNT/A, which might be at least partially responsible for its analgesic effect. (**B**) Peripheral administration of BoNT/A reduced spinal microglial, but not astroglial, activation after sciatic nerve injury. (**C**,**D**) The data obtained from in vitro studies revealed that BoNT/A can directly influence microglial cells and it is achieved through the modulation of TLR2 receptor, SNARE proteins and intracellular pathways in microglial cells. BoNT/A diminishes LPS-induced phosphorylation of p38, ERK1/2, and NF-κB and reduces the release of pro-inflammatory factors, such as IL-1β, IL-18, IL-6, and anti-inflammatory IL-10 in microglia. No effects of BoNT/A on astroglia were observed. Mechanism of BoNT/A in glial cells is related to activation of TLRs, type 2 and 4. Complete activation of TLR2 in astroglia requires the presence of the microglial TLR4 receptor. Glial cross-talk may explain the lack of effect of BoNT/A on astroglia and it was suggested that the molecular target of BoNT/A is TLR2. See detailed description in the text **Abbreviations:** SNAP, synaptosomal-associated protein; TLR, Toll-like receptor; MyD, myeloid differentiation primary response gene; ERK1/2, extracellular-signal-regulated kinase 1/2; NF-κB, nuclear factor-κB; NOS2, inducible nitric oxide synthase; IL, interleukin; LPS, lipopolysaccharide, BoNT/A, botulinum toxin serotype A.

In a 2016 paper by Zychowska et al. [39], intraplantar injections of BoNT/A not only relieved neuropathic pain-related behaviours but also restored the neuroimmune balance disturbed after nerve injury. BoNT/A diminished CCI-induced level of IL-1β and IL-18 within the spinal cord and/or the DRG, and in parallel, it enhanced the levels of the anti-nociceptive interleukins IL-1RA and IL-10. Obtained data suggest that BoNT/A, in addition to altering neuronal function, can also influence spinal microglial cells [38,39]; however, it is still unclear whether those BoNT/A actions are mediated in a direct or indirect manner. The latest in vitro research by Piotrowska et al. 2017 [62] shed new light on the analgesic effect of BoNT/A and suggested a possible direct impact of this toxin on microglia in the CNS (Figure 1).

## 3. Far Beyond the Neurons—The Role of Glial Cells in Bont/A-Induced Analgesia

BoNT/A affects both SNAP-25 and -23 in neuronal cells; however, it was recently suggested that BoNT/A is also able to cleave SNAPs in astroglia and microglia [63,64]. Within the CNS, glial cells seem to play a crucial role in neuronal homeostasis [2,4,65,66]. Two main types of glial cells are present: macroglia (which include astroglia, oligodendrocytes and radial cells, including Bergmann and Müller cells) and microglia. Under physiological conditions, glial cells account for 70% of cells, and resting microglia account for only 5–20% of cells [3]. It is now obvious that glial cells are an important component of neural tissue and play a crucial role in the synthesis, release and uptake of many factors. The contribution of astroglia and microglia in the progression of neuropathy is well known [67,68]; however, the role of other macroglial cells (oligodendrocytes and radial cells) is still not well established. Under neuropathic pain, activated astroglia and microglia play a role in synaptic transmission based on the presence of similar receptors, ion channels, transporters and intracellular signaling cascades to neurons [66,68]. Glial cells are also capable of conducting active communication with neighboring neurons by gap junctions [69,70,71] and synapses [72,73,74].

Astroglia represent the most abundant cell population in the nervous system and are critical in maintaining the homeostasis of their surrounding environment by regulating the concentrations of the neurotransmitters, ions, and proteins in the synaptic cleft. Activation of astroglia as a result of peripheral neuropathy occurs approximately four days after microglial activation and persists until 12 weeks or longer after the injury, thus suggesting that astroglia are involved in the persistence of pain [68,75,76]. The direct influence of BoNT/A on astroglia was unclear, since the study by Parpura et al. (1995) [64] showed the expression of some of the SNARE protein complexes but not that of SNAP-25. The authors analyzed post-nuclear astroglial cell membrane extract, not the whole lysates as in Piotrowska et al. 2017 [62]. The authors, using in vitro primary cell culture studies, have demonstrated that astroglial cells possess both mRNA and protein for the SNAREs SNAP-25 and SNAP-23 [62]. These data correlate well with the study of Marinelli et al. (2012) [61] showing that BoNT/A exerts analgesic effects on neuropathic pain through the cleavage of SNAP-25 in spinal astroglia. Interestingly, in vitro studies gave evidence that BoNT/A did not influence pro-nociceptive factors (IL-1β, IL-6, IL-18, and NOS2) or anti-nociceptive factors (IL-1RA, IL-10, and IL-18BP) in LPS-stimulated astroglial cell cultures [62] (Figure 1). The authors revealed that BoNT/A does not affect the activation of MAPKs, p38 and ERK1/2, or NF-κB pathways in LPS-treated primary astroglial cell cultures. Moreover, they showed no changes in TLR2 and TLR4 expression, the adaptors for which initiate the activation of NF-κB and MAPK cascades in astroglia, which is required for the production of nociceptive factors [77]. Surprisingly, BoNT/A appears to have no more than a slight direct effect on astroglia. 

Recently, some authors have suggested that the molecular mechanism of BoNT/A action involves microglia [38,41]. Microglial cells are highly dynamic immune cells, which are responsible for the maintenance of homeostasis in the CNS [66,78]. They are known to dynamically modulate neuronal functions under neuropathic pain. They are the first cell type to become spinally activated following peripheral nerve injury [76] and remain active for several weeks [66,79,80,81]. Several reports have provided evidence that inhibitors of microglial activation, such as minocycline, propentofylline, and pentoxifylline, might largely limit the development of neuropathic pain [38,51,78,82,83,84] and those effects are due to reduced activation of microglial cells, which entails the inhibition of numerous cytokine secretion [85,86,87]. In 2011, Mika et al. [38] showed that a single intraplantar administration of BoNT/A, after nerve injury, attenuated neuropathic pain-related behaviors in neuropathic rats and, in parallel, reduced spinal microglial activation. The results provide evidence that BoNT/A, in addition to having an impact on neuronal functions, can also influence the activation of microglia; therefore, the involvement of these non-neuronal cells in BoNT/A action should also be regarded. Although in vivo studies have shown that BoNT/A influences the activation of microglial cells in rat and mouse neuropathic pain models [38,41], it was not clear if that occurred in a direct or indirect manner. The well-characterized molecular targets of BoNT/A action are superfamily of SNARE proteins. The studies of Hepp et al. 1999 [63] suggested that in microglia, SNAP-25 is replaced by SNAP-23. SNAP-23 is structurally and functionally similar to SNAP-25 and binds tightly to multiple syntaxins and synaptobrevins/VAMPs. It is a crucial component of the high-affinity receptor for the general membrane fusion machinery and is an important regulator of transport vesicle docking and fusion. In 2017, Piotrowska et al. [62] using in vitro primary cell culture, demonstrated the presence of both mRNA and protein for SNAP-23, but not for SNAP-25, in microglia. The in vitro studies correspond well with in vivo data obtained by Marinelli et al. (2012) [61], where the authors did not observe staining for cleaved SNAP-25 protein co-localized with microglia. Therefore, it seems that SNAP-23, not SNAP-25, plays an important role in the effects of BoNT/A on microglia.

Zychowska et al. (2016) [39] showed that BoNT/A injection reverts the neuro-immune changes after sciatic nerve injury. In vitro studies with glial cell cultures [62] revealed the inhibitory action of BoNT/A on the intracellular cascades, which is possibly involved in changes in the expression of many nociceptive factors. BoNT/A prevents the LPS-induced upregulation of pro-nociceptive factors (IL-1β, IL-18, NOS2) through the modulation of intracellular pathways activation (NF-κB, p38 and ERK1/2) and increases the expression of TLR2 and its adaptor protein MyD88 in microglia (Figure 1). These results can be compared with those published in 2015 by Kim et al. [88] who showed that BoNT/A inhibited LPS-upregulated NO production in RAW264.7 macrophages by blocking the activation of ERK and p38.

Recently, many papers have shown that the inhibition of the MAPK family members (namely p38 and ERK1/2) leads to lower rates of neuropathy in animal models, down-regulated pro-nociceptive factors and enhanced opioid efficiency [84,89,90,91,92,93,94,95,96]. The in vitro results of Piotrowska et al. (2017) have shown that BoNT/A decreased the LPS-induced activation of p38 and ERK1/2 in microglia; similar results were obtained in the monocyte/macrophage cell line RAW264.7 by Kim et al. (2015) [88]. Many studies have shown an important role for the NF-κB pathway in nociception and microglial cell activation [94,95,96,97,98,99]. In vitro results of Piotrowska et al. (2017) [62] revealed that BoNT/A reduced NF-κB phosphorylation in LPS-treated microglia. It has been shown that the inhibition of NF-κB with a potent inhibitor, parthenolide, diminished the symptoms of neuropathic pain, and, moreover, it potentiated morphine-induced analgesia and reduced the levels of pro-nociceptive factors produced by microglial cells (such as IL-1β, IL-18, NOS2) [95].

Among the numerous receptors expressed by microglial cells, the family of TLR receptors, especially subtypes 2 and 4, represents a possible link between microglia activation and nerve injury and illustrate an essential role of those cells in the development of neuropathy [100,101,102,103]. The TLRs are a type of receptor, which are important for pathogen recognition. Activation of those receptors leads to the initiation of direct antimicrobial pathways, expression of co-stimulatory factors, and release of cytokines via NF-κB and/or MAPK signaling pathways. The TLRs family recognize pathogen-associated molecular patterns (PAMPs), expression of which is characteristic for infectious agents [104,105]. The TLRs can also discern danger-associated molecular patterns (DAMPs), products of nerve injury [106]. It has been confirmed that TLR2 or TLR4 knockout mice have less microglial activation after nerve injury and fewer neuropathic pain symptoms [100,101,103,107]. Piotrowska et al. (2017) [62] revealed that LPS induced a decrease in TLR4 in microglia. Recently, studies using macrophages revealed that BoNT/A is sensed by TLR2, but not by TLR4 [88]. In turn, BoNT/A increased the TLR2 level in LPS-stimulated microglia. It has been demonstrated that TLR4 activation is mediated by dimerization of adaptor proteins (MyD88 or TRIF), but TLR2 uses only MyD88 [108]. Our data have proven that BoNT/A administration rescues downregulated by LPS level of MyD88 in microglia [62]. Recently, an interaction between TLR signaling and SNARE proteins has been suggested. In 2014, Nair-Gupta et al. [109] showed that MyD88-dependent TLR signaling is involved in the phosphorylation of SNAP-23 present on the phagosome in dendritic cells. The phosphorylated SNAP-23 protein stabilizes SNARE complexes, which leads to fusion with the endosomal recycling compartment and ultimately to cross-presentation. In our opinion, we observed a similar phenomenon in microglia: these cells act as key players in the immune response in the CNS. Microglia are the resident innate immune cells and within CNS they are responsible for the early control of infections and for the recruitment of cells of the adaptive immune system, which are required for pathogen clearance [110]. Thus, it seems that the microglial TLR/MyD88/NF-κB cascade contributes to the decrease of SNAP-23. Recently, it has been suggested that microglia are characterized by heightened expression of TLRs and by a stronger response to LPS compared to astroglia [111]. Furthermore, in 2012 Holm et al. [111] discovered that the response of astroglial cells to TLR2 agonists is completely dependent on the presence of functional TLR4 in microglial cells. The activation of TLR4 by LPS induces a synthesis of closely related TLR2 [112]. In 2017, Piotrowska et al. [62] did not observe changes in expression of the analyzed factors, with the exception of SNAP-23 and SNAP-25, after BoNT/A treatment in LPS-stimulated astroglia. The authors suggested that TLR2 is another important molecular target for BoNT/A. In sum, BoNT/A exerts its anti-inflammatory action by inhibiting signaling pathways (such as NF-κB, p38 and ERK1/2) activation in microglia; very importantly, it can directly interact with TLR2. Based on the current state of knowledge, we can hypothesize that the full activation of astroglial TLR2 requires the presence of functional TLR4 in microglia, which emphasizes the significant cross-talk between those cells types within the CNS. 

## 4. Conclusions

Further investigation concerning the action of BoNT/A in the CNS is needed and will provide priceless information to help understand the pathophysiology of neuropathic pain. The literature provides evidence that BoNT/A, in addition to having an impact on neuronal functions, can also influence the activation of microglia; therefore, the involvement of these non-neuronal cells in the BoNT/A mechanism of action should also be regarded as an important component of its analgesic effects. However, BoNT/A appears to have no more than a slight direct effect on astroglia. In our opinion, BoNT/A is a powerful modulator of neuron–glia interactions in the CNS in the context of neuropathic pain. More research into BoNT/A as a treatment for neuropathy is warranted because it could be an attractive alternative for patients who do not respond positively to other drugs.
